# Novel Biallelic PLEKHG5 Variant Associated With Intermediate Charcot‐Marie‐Tooth Disease: Case Report From South America

**DOI:** 10.1111/jns.70099

**Published:** 2026-01-21

**Authors:** Rafael Oliveira Vidon, Pedro José Tomaselli, Caroline Bittar‐Braune, Izabela Jardim Pitta, Glenda Corrêa Borges de Lacerda, Márcia Jardim

**Affiliations:** ^1^ Neurology Department Hospital Universitário Gaffrée e Guinle, Universidade Federal Do Estado Do Rio de Janeiro (UNIRIO) Rio de Janeiro Brazil; ^2^ Department of Neurosciences and Behaviour Sciences School of Medicine of Ribeirão Preto, University of São Paulo Ribeirão Preto Brazil; ^3^ Neurology Department Hospital Universitário Antônio Pedro, Universidade Federal Fluminense (UFF) Niterói Brazil; ^4^ Hospital Universitário Pedro Ernesto, Universidade do Estado do Rio de Janeiro (UERJ), and Oswaldo Cruz Foundation (Fiocruz) Rio de Janeiro Brazil

**Keywords:** Charcot‐Marie‐tooth disease, hereditary neuropathy, intermediate CMT, PLEKHG5

## Abstract

**Background and Aims:**

Biallelic pathogenic variants in *PLEKHG5* are associated with two distinct recessive phenotypes, including distal hereditary motor neuropathy AR type 4 and intermediate Charcot‐Marie‐Tooth disease type C (CMT). No South American cases have been previously reported.

**Methods:**

We evaluated a male patient with suspected hereditary neuropathy using clinical, electrophysiological, and genetic studies.

**Results:**

Symptoms began at 12 years with progressive distal weakness. At 40 years, he had foot drop, pes cavus, distal atrophy, areflexia, and sensory loss to the knees. Disability scales indicated moderate impairment. Electroneuromyography revealed abolished responses in the lower limbs and motor conduction velocities in the intermediate range (35–40 m/s). Genetic analysis identified the homozygous variant c.59G>A (p.Arg20Gln) in *PLEKHG5*, currently classified as VUS.

**Interpretation:**

This reports presents a case from South American linking a homozygous *PLEKHG5* variant to recessive intermediate CMT, expanding the geographic and phenotypic spectrum of *PLEKHG5*‐related neuropathies.

## 1 Introduction

Biallelic pathogenic variants in the PLEKHG5 gene have been implicated in distal hereditary motor neuronopathy type 4 (dHMN4) [1] and autosomal recessive intermediate Charcot‐Marie‐Tooth disease type C (CMTRIC) [2]. Reported cases remain rare, but collectively they define a clinical continuum in which phenotypic expression ranges from predominantly distal, sensorimotor neuropathies to more severe, proximal motor phenotypes resembling spinal muscular atrophy. Intermediate CMT typically presents with distal weakness and mild sensory involvement, whereas dHMN4 is characterized by earlier onset, proximal weakness, scapular winging, early ambulation loss, and contractures, usually without sensory abnormalities.

Importantly, identical PLEKHG5 variants may produce divergent phenotypes, even within the same family, underscoring the absence of a strict genotype–phenotype correlation. Frameshift, missense, and splice‐site variants have been reported in both homozygous and compound heterozygous states, with most cases identified in populations with a high prevalence of consanguinity. Collectively, these observations position PLEKHG5‐related neuropathies as part of a pleiotropic spectrum, in which the main discriminators are the distribution and severity of weakness and the presence or absence of sensory involvement.

Here we describe a Brazilian patient carrying a homozygous PLEKHG5 variant associated with recessive intermediate CMT, thereby extending the geographic distribution of reported cases and contributing to the ongoing delineation of the PLEKHG5‐related spectrum.

## 2 Materials and Methods

The proband underwent clinical, electrophysiological, imaging, and laboratory investigations as part of his diagnostic evaluation. Genetic analysis was performed using a targeted next‐generation sequencing (NGS) multigene panel for hereditary neuropathies. All procedures followed institutional ethics approval, and informed consent was obtained.

## 3 Results

### 3.1 Clinical Features

The patient was the third child of healthy parents from a family with a tradition of consanguineous marriage; his parents were first‐degree cousins. Early neurodevelopment was normal, with no reported delay in achieving independent walking. From 12 years of age, he developed gait difficulties with frequent falls and ankle sprains, progressing to bilateral foot drop by age 16. At 22, based on progression and family history, he was clinically diagnosed with hereditary neuropathy. By age 30, he reported distal paresthesia in a stocking distribution up to the knees.

At age 40, neurological examination revealed a steppage gait, marked distal leg atrophy, ankle dorsiflexion weakness (MRC 2/5), knee extension weakness (MRC 4/5), and preserved proximal and upper limb strength (MRC 5/5). Deep tendon reflexes were globally absent. Sensory testing showed normal upper‐limb findings and distal hypoesthesia in the lower limbs. Cranial nerve examination was normal. Functional scores were: CMT Examination Score (CMTESv2) of 10, CMT neuropathy score (CMTNSv2) of 13, Neuropathy Impairment Score—Lower Limbs (NIS‐LL) 50.5, and Polyneuropathy Disability Score (PND) 2.

### 3.2 Laboratory, Neurophysiology, and Genetic Testing

Routine laboratory evaluation was unremarkable except for a mild elevation of creatine phosphokinase (436 U/L), 2.3‐fold upper limit of normal (ULN). No scoliosis was noted.

Nerve conduction studies demonstrated a length‐dependent sensorimotor neuropathy. Sensory nerve action potentials were absent in the lower limbs and reduced in amplitude in the upper limbs. Motor conduction velocities in the upper limbs were within the intermediate range (35–40 m/s). F‐wave latencies were mildly prolonged (~1.4‐fold the upper limit of normal), most prominently in the median nerves (Table 1).

**TABLE 1:** Nerve conduction data.

		Left/right	Normal values
Motor	Median		
	Distal latency	5.0/4.8 ms	≤ 4.1
	Distal CMAP	7.1/9.4 mV	> 4.0 mV
	VCM	40.0/35.4 m/s	≥ 49 m/s
	Ulnar		
	Distal latency	3.8/3.5 ms	≤ 3.3 ms
	Distal CMAP	4.6/7.4 mV	≥ 6 mV
	VCM	41.3 (elbow 35.8)/36.7 (elbow 36.2)	≥ 49 m/s
	Fibular		
	Distal latency	Absent	6 ms
	Distal CMAP	Absent	2.8 mV
	VCM	Absent	44 m/s
	Tibial		
	Distal latency	Absent	6 ms
	Distal CMAP	Absent	2.8 mV
	VCM	Absent	44 m/s
Sensitive	Median		
	SNAP	6.8/10.8 μV	≥ 9 μV
	VCS	45.1/46.6 m/s	≥ 50 m/s
	Ulnar		
	SNAP	2.0/3.4 μV	≥ 9 μV
	VCS	41.5/41.5 m/s	≥ 50 m/s
	Radial		
	SNAP	2.8/3.6 μV	≥ 9 μV
	VCS	41.6/35.7 m/s	≥ 50 m/s
	Sural		
	SNAP	Absent	≥ 6 μV
	VCS	Absent	≥ 40 m/s
F waves			
	Median	44/44.4 ms	> 31 ms

Abbreviations: CMAP, compound motor action potential; MCV, motor conduction velocity; SCV, sensory conduction velocity; SNAP, sensory nerve action potential.

NGS neuropathy panel identified a homozygous PLEKHG5 variant, NM_014799.4:c.59G>A (p.Arg20Gln), classified as a variant of uncertain significance (VUS) by ACMG criteria.

## 4 Discussion

This case represents a report from Brazil of recessive intermediate CMT associated with PLEKHG5. Pathogenic variants in this gene were first linked to childhood‐onset SMA [1] and later to recessive polyneuropathies [2]. Subsequent reports expanded the spectrum to include dHMN, intermediate and axonal CMT [2, 3, 4, 5, 6], and SMA‐like presentations [3, 4, 5].

The variability of PLEKHG5‐related phenotypes is striking. Initial descriptions emphasized severe proximal and distal weakness with SMA‐like features but no sensory loss. Later studies identified intermediate CMT with modest sensory involvement and conduction velocities in the intermediate range, as well as mixed phenotypes with scapular winging or atypical features. Collectively, these observations reinforce the pleiotropic nature of PLEKHG5, in which clinical expression extends across a continuum from pure motor neuron disease to classical sensorimotor CMT.

Our patient carries the c.59G>A (p.Arg20Gln) missense variant in the N‐terminal region containing the RhoGEF domain, a region essential for the regulation of motor neuron autophagy and synaptic vesicle turnover [7].

Perturbation of this pathway may promote axonal degeneration through vesicle accumulation. Although formally classified as a VUS, several factors argue for pathogenicity: its rarity, the homozygous state in a consanguineous pedigree, and concordance with reported intermediate CMT phenotypes.

Epidemiologically, PLEKHG5 mutations are most often described in consanguineous populations of the Middle East, North Africa, and Southern Europe, with additional cases in South and East Asia [2]. The scarcity of reports in Latin America may reflect both true rarity and underdiagnosis due to limited access to genetic testing and clinical overlap with SMA and dHMN.

In conclusion, this case broadens the geographic range of PLEKHG5‐associated neuropathies and underscores the need for systematic genetic testing in atypical neuropathies. It contributes to refining the intermediate CMT phenotype and highlights the absence of strict genotype–phenotype correlation, supporting the view of PLEKHG5‐related disorders as a continuous clinical spectrum.

## Conflicts of Interest

The authors declare no conflicts of interest.

## Data Availability

The data that support the findings of this study are available from the corresponding author upon reasonable request.
